# Developing a sensor-based mobile application for in-home frailty assessment: a qualitative study

**DOI:** 10.1186/s12877-021-02041-z

**Published:** 2021-02-04

**Authors:** Marcela D. Blinka, Brian Buta, Kevin D. Bader, Casey Hanley, Nancy L. Schoenborn, Matthew McNabney, Qian-Li Xue

**Affiliations:** 1 Center on Aging and Health, Johns Hopkins University, 2024 E. Monument Street, Suite 2-700, Baltimore, MD 21205 USA; 2 Division of Geriatric Medicine and Gerontology, Johns Hopkins University School of Medicine, Baltimore, MD USA; 3 The Johns Hopkins University Applied Physics Laboratory, Laurel, MD USA

**Keywords:** Frailty, Wearable, Health services

## Abstract

**Background:**

Frailty syndrome disproportionately affects older people, including 15% of non-nursing home population, and is known to be a strong predictor of poor health outcomes. There is a growing interest in incorporating frailty assessment into research and clinical practice, which may provide an opportunity to improve in home frailty assessment and improve doctor patient communication.

**Methods:**

We conducted focus groups discussions to solicit input from older adult care recipients (non-frail, pre-frail, and frail), their informal caregivers, and medical providers about their preferences to tailor a mobile app to measure frailty in the home using sensor based technologies. Focus groups were recorded, transcribed, and analyzed thematically.

**Results:**

We identified three major themes: 1) perspectives of frailty; 2) perceptions of home based sensors; and 3) data management concerns. These relate to the participants’ insight, attitudes and concerns about having sensor-based technology to measure frailty in the home. Our qualitative findings indicate that knowing frailty status is important and useful and would allow older adults to remain independent longer. Participants also noted concerns with data management and the hope that this technology would not replace in-person visits with their healthcare provider.

**Conclusions:**

This study found that study participants of each frailty status expressed high interest and acceptance of sensor-based technologies. Based on the qualitative findings of this study, sensor-based technologies show promise for frailty assessment of older adults with care needs. The main concerns identified related to the volume of data collected and strategies for responsible and secure transfer, reporting, and distillation of data into useful and timely care information. Sensor-based technologies should be piloted for feasibility and utility. This will inform the larger goal of helping older adults to maintain independence while tracking potential health declines, especially among the most vulnerable, for early detection and intervention. Keywords: Frailty, wearable, health services.

**Supplementary Information:**

The online version contains supplementary material available at 10.1186/s12877-021-02041-z.

## Background

The aging of the world’s population is expected to increase dramatically in coming decades. Between 2015 and 2050, the global percentage of adults ages 65 years will nearly double from 8.5 to 16.7% [1]. Given this demographic shift, a major challenge of healthcare worldwide is providing medical care for vulnerable older adults with complex health problems. A common condition among geriatric patients is the frailty syndrome, which affects 15% of non-nursing home residents aged 65 and older in the US [2] and up to 27.3% globally [3]. Physical frailty manifests as a state of vulnerability to adverse health outcomes, associated with disability, hospitalization, institutionalization, and mortality, and is hypothesized to result from dysregulation across multiple physiological systems [4]. A consensus statement by an international group of researchers proposed that adults 70 years or older should be screened for physical frailty, and that the frailty diagnosis can guide clinical care planning [5].

Recommendations for strategies to prevent and manage frailty, and the concurrent push to have older adults “age in place” [6], have increased the need to find effective and user-friendly approaches for early identification of frailty and for effective communication about frailty prevention and treatment [7]. Interest in incorporating frailty assessment in research and clinical practice has accelerated, along with frequent calls to simplify assessments (e.g., substituting walking speed assessments with self-reported walking difficulty) so as to streamline patient evaluations in clinical practice. However, there are limits to what assessments can be feasibly and consistently done in the clinical setting given time and resource constraints, compounded by a shortage in the geriatrician workforce [8]. However, studies from us and others reported notable discrepancies in frailty classification when simplified measures of frailty were used to promote speed and ease of patient evaluation in clinical practice [9, 10]. The resulting misclassification may both hamper the discovery of frailty biology and risk harming patients due to misdiagnosis and mistreatment.

The development of new sensor-based mobile technologies provides an opportunity to bridge the gap between the desire to have vulnerable older people age in place, and still meet their medical needs. Technology, chiefly sensor technology in combination with mobile applications, may be particularly well suited to facilitate and simplify in-home assessment of frailty and its progression in frail older adults, without sacrificing diagnostic accuracy. This can be accompanied by enabling real-time data feeds from the patient to improve patient-caregiver-doctor communication.

There is limited knowledge on patients’, their caregivers’, and medical providers’ perspectives on the use of sensor technology for in-home frailty assessment; and how its results should be communicated and used to aid clinical decision-making has not been investigated. This qualitative focus group study explores the perspectives and preferences of older adult care recipients across the frailty spectrum, their informal caregivers, and geriatric medicine medical providers, regarding the use of wearable and/or installed sensors to assess frailty at home. The ability to harness technology to better identify at-risk individuals, and provide opportunities to intervene during the early manifestations of frailty, is particularly important when reversal may be possible. The ultimate goal is to facilitate more efficient approaches to the care of older adults toward a proactive and preventive model.

## Methods

### Participant recruitment

Care recipients: Older adults were recruited from a registry of adults ages 65 and older, who had previously given permission to be contacted for studies for which they may be eligible (Johns Hopkins Medicine IRB# NA 00013162) and whose frailty status had been assessed within the last 3 years. The registry consist of volunteers recruited from two outpatient clinics, as well as volunteers from the Baltimore metropolitan area who answered newspaper advertisements. Potential participants were contacted by phone to assess the following eligibility criteria: aged 65 years or older; English-speaking; able to provide informed consent to the study; and receiving help, at least once a week, with chores such as shopping, paying bills, managing medication, preparing a meal, walking, dressing, bathing from an informal and unpaid caregiver. The last one was added for the purpose of recruiting caregivers.

**Fig. 1:**
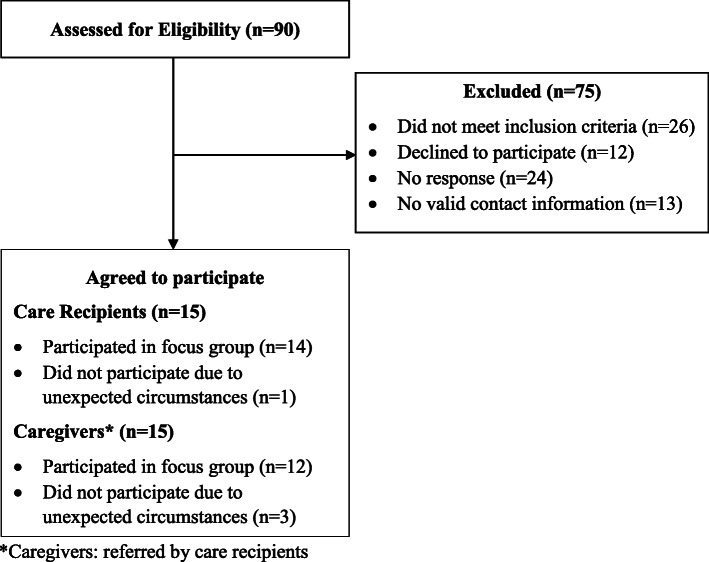
Participant Recruitment/Enrollment

Caregivers: once care recipients met the eligibility criteria they were then asked to share the study information with their informal caregiver. Caregivers who agreed to be contacted by a study team member, provided unpaid assistance regularly with activities of daily living (ADLs) or instrumental activities of daily living (IADLs) were eligible to participate. Subsequently, care recipients and caregivers were invited to participate in the focus groups (see Fig. 1).

Medical providers were recruited from two sources: providers (physicians, nurses, physical therapists, occupational therapists) from the Hopkins Elder Plus (HEP) Day Healthcare Center based at Johns Hopkins, and medical providers from the Division of Geriatric and Gerontology at Johns Hopkins. HEP is a Program of All-inclusive Care for the Elderly (PACE), within a network of over 130 PACE programs in the US [11]. A primary objective of PACE is to keep nursing home-eligible older adults living in the community. Medical providers from the Division of Geriatric and Gerontology at Johns Hopkins included geriatricians, nurses, physical therapists, occupational therapist, medical assistant, and program administrator. Any provider from these programs who agreed to participate in the study were accepted as participants.

### Frailty assessment

A physical frailty phenotype assessment was performed to verify the frailty status of the care recipients prior to the focus group sessions if the previous assessment had been more than 12 months prior. The assessment included five binary criteria coded as whether present or absent: weight loss, weakness, exhaustion, slowness, and low physical activity [4, 12]. Participants were defined as frail if three or more of the five criteria were present; pre-frail if one or two criteria were present; and non-frail if no criteria were present. Scores were calculated using the Hopkins Frailty Assessment Calculator [13].

Focus Groups

Eight focus group sessions were held to solicit input from older adult care recipients (3 focus groups), their informal caregivers (3 focus groups), and medical providers’ perspectives (2 focus groups) about using sensor-based technologies in the home. Older adults care recipients and caregivers were respectively stratified into separate focus groups based on frailty status of the older adults (non-frail, pre-frail, and frail). Providers were stratified by the clinical programs from which they were recruited. Written informed consent was obtained as described in the institutional review board protocol. Basic demographic data and data describing the amount of time caregivers spent providing care were collected. Each of the focus groups took place in a conference room, with participants seated around a table with facilitators comprised of a study coordinator, principal investigator, and another research team member.

The focus group sessions began with the facilitator providing a definition of frailty to stimulate the discussion. At the beginning of each focus group session, participants viewed a short video showing how different sensors can be used in the home to monitor and individual’s health. Different interview guides were used to facilitate discussion depending on type of participant in the particular focus group (i.e., older adult, caregiver, or provider), as the perspective of each group (e.g., with respect to knowledge of frailty status) was expected to be different. All interview guides were developed for this study and are included in the Additional file 1. For example, the interview guide questions related to knowledge of the frailty status of a patient were different for providers (e.g. … would knowing the risks of frailty be helpful to you as a medical professional?) compared to older adults and caregivers (e.g. would knowing the risks of frailty make someone more motivated to make a positive change about their health?). Participants were encouraged to explore various technology-related topics including device selection (e.g., wrist-, arm-, abdomen-, waist-, leg-, hip-, neck-, and ankle-worn devise options), usability, adoptability, privacy concerns, data security, duration and frequency of monitoring, how interactive users want to be with the technology, and factors that could modify and influence their perspectives and preferences. As the development of actual sensors will be the subject of future work, in this study, we did not specifically assess participants’ views of specific sensor concepts.

### Coding and analyses

Audio recordings of each focus group were transcribed verbatim and analyzed for thematic content using the Atlas.ti software program used in qualitative data analysis [14, 15]. Codes for each focus group were developed empirically, without a pre-determined coding scheme. The data were analyzed per focus group category (older adults, caregivers, providers) by the study coordinator and verified by a second team member. Following open coding procedures, themes and subthemes were identified and entered into a codebook. The codebook included a definition for each theme and subtheme, as well as representative quotes from the focus groups’ participants. The codes identified in the transcripts of earlier focus groups were used to assist with the coding of subsequent focus groups. Coding consensus was reached through discussion among the team members [16].

## Results

### Participant characteristics

Ninety potential participants were contacted for the study and 15 care-recipients and their caregivers agreed to participate. Due to health and transportation problems, one care-recipient and three caregivers were unable to participate. The mean age for care recipients was 76 (standard deviation (sd) = 7). Most were Caucasian (71%), female (64%), and reported being in good or better health (43%). Similarly, caregivers were mostly Caucasian (75%) and female (67%), with a mean age of 68 (sd = 8). Assistance provided by caregivers included cooking, shopping, physical support (e.g. walking), household chores and driving to doctors’ visits. Additional participant characteristics are provided in Table 1. Medical providers were mostly female, nurse practitioners, registered nurses, or licensed practical nurses (*n* = 6), followed by physical and occupational therapists and support staff (*n* = 5), and geriatricians (*n* = 3). All were involved with the care of older adults.

**Table 1:** Participant characteristics

Item	Care Recipient			Caregiver
	Overall *n* = 14	Non Frail/Pre-frail *n* = 8	Frail n = 6	*n* = 12
Age: mean (sd)	76.1 (6.5)	73.9 (5.5)	79.2 (6.9)	68.2 (8.0)
Sex: % female	64.3%	62.5%	66.7%	66.7%
Race: % Caucasian	71.4%	80%	20%	75%
Education: %				
High School/Equivalent	21.4%	25.0%	16.7%	41.7%
College	28.6%	37.5%	16.7%	33.3%
Post graduate	50.0%	37.5%	66.7%	25.0%
Living Status: %				66.7%^a^
Alone	35.7%	12.5%	66.7%	
Spouse/partner	50.0%	62.5%	33.3%	
Son/daughter(s)	14.3%	25.0%	0%	
Care Recipient Health Status: %				
Excellent	14.3%	25.0%	0%	8.3%
Very good	21.4%	37.5%	0%	25.0%
Good	42.9%	25.0%	66.7%	25.0%
Fair	0%	0%	0%	33.3%
Poor	21.4%	12.5%	33.3%	8.3%

^a^Living with care-recipient

Three key themes emerged from the focus groups common to all three types of participants, including perspectives on frailty, acceptance of home-based sensors, and data management concerns. A summary of theme contribution by type of participant is provided in Table 2.

**Table 2:** Theme contribution by type of participant

Themes	Non-Frail (*N* = 10)		Pre-Frail (*N* = 9)		Frail (*N* = 7)		
	CR (*N* = 5)	CG (*N* = 5)	CR (*N* = 5)	CG (*N* = 4)	CR (*N* = 4)	CG (*N* = 3)	Providers (*N* = 14)
**Theme 1: Perspectives on frailty**							
Subtheme 1: Frailty is declining physical function, not disability	80% (4)	60% (3)	40% (2)	75% (3)	100% (4)	100% (3)	79% (11)
Subtheme 2: Knowing Frailty Status Is Important and Useful	60% (3)	100% (5)	80% (4)	100% (4)	75% (3)	67% (2)	64% (9)
**Theme 2: Perceptions of Home-Based Sensors**							
Subtheme 1: Benefits of Home-Based Sensors	60% (3)	60% (3)	40% (2)	100% (4)	100% (4)	67% (2)	57% (8)
Subtheme 2: Concerns about Home-Based Sensors	60% (3)	80% (4)	80% (4)	75% (3)	75% (3)	67% (2)	50% (7)
**Theme 3: Data Management Concerns**							
Subtheme 1: Concerns about data interpretation, reporting and responsibility	60% (3)	40% (2)	80% (4)	75% (3)	75% (3)	100% (3)	50% (7)
Subtheme 2: Data security	40% (2)	60% (3)	80% (4)	50% (2)	75% (3)	67% (2)	36% (5)

*CR* care recipient, *CG* caregiver

#### Perspectives on frailty

Generally, focus group participants (care recipients, caregivers, and providers) had similar perspectives and understanding of frailty as a decline in an older adult’s physical function over an extended period of time, as distinct from their level of physical ability (or disability). All three groups identified attributes of physical decline in frail older adults, as well as declines in their ability to recover or respond to life events.

##### Subtheme 1: frailty is declining physical function, not disability

Care-recipients, caregivers, and providers all identified physical attributes of frailty in older adults including unintentional weight loss, loss of muscle mass and strength, loss of agility, loss of mobility, decreased gait speed, and loss of resiliency.

A frail care recipient noted that *“I’m tired a lot in general, things like that. So I never really thought of myself as being frail to whatever that description and it’s like, oh, no, that’s me…”.*

A provider defined a frail individual as*“… someone who’s lost weight and is just very, very thin; in the physical context would be, you know, people thin, weaker, things like that”* whereas an example of a disabled person is “*an athlete who’s in a wheelchair who plays basketball, that person’s disabled but certainly far from my imagination of being frail.”*

##### Subtheme 2: knowing frailty status is important and useful

Caregivers and care-recipients both felt that knowing the older adult’s frailty status would facilitate making lifestyle and home life changes, such as making plans about how to handle their declining health. For care-recipients and caregivers, this meant changes in lifestyle and avoidance of risky behaviors and situations. As explained by a care recipient, knowledge of their frailty status could mean: “*[c]hang[ing] your lifestyle, adapt to -if you have – if you live in a house with lots of stairs maybe move to a one-floor house or something … lowering the risk[s] that come with frailty.*”

There was a consensus among participants that safety and harm reduction are important factors for appropriately managing frailty, although their perspectives were somewhat different based on their different roles. Providers indicated that knowing a patient’s frailty status was useful in developing an appropriate treatment plan and in making care decisions that affect quality of life, as well as reducing the negative consequences associated with treatment. For example, one provider explained “*harm reduction … becomes really important, too. So whether that [would] be … watching whatever medications we’re prescribing or making sure they have appropriate equipment [so] that if they fall they don’t hurt themselves.*”

#### Perceptions of home-based sensors

Providers, care recipients, and caregivers expressed both positive and negative views of home-based sensors.

##### Subtheme 1: benefits of home-based sensors

Providers, care-recipients and caregivers felt that sensors would be useful to provide objective data and would be helpful and cost-effective way to track changes associated with frailty and declining health. They also anticipated that sensors could reduce health-care costs, which would be good for the patient, caregiver and provider, and would enhance the independence of older adults. As one care recipient stated, “*the reason this is being done is that it saves … money. … So the more independent we are the happier everybody is, and the cheaper it is for everybody else.*” A provider indicated that home sensors could be useful because *“sometimes we’ll admit people to facilities to see if they’re having behaviors or, you know, they’ll describe being up all night going to the bathroom.”*

##### Subtheme 2: concerns about home-based sensors

Providers identified concerns about affordability and access for some of their patients. As one provider explained, *“If it involves an Internet connection, a number of our participants do not have Internet and can’t afford it. “*A main concern for care recipients was care quality rather than access. Care recipients worried that the use of sensors would tend to replace or reduce in-person visits. One care recipient stated “*My concern about these kinds of systems are that you don’t lose the personal contact.”* Caregivers focused on the potential for loss of autonomy of care recipients. One caregiver stated that *“…it sounds like big brother looking at you.”*

#### Data management concerns

Care-recipients, caregivers, and providers shared concerns about the large volume of data generated and whether the data could be interpreted and reported in a timely and useful manner. Additionally, providers had unique concerns regarding responsibility for data administration and reporting (including to whom the data would be reported), and how such data could be useful in preparing overall care plans for older adults.

##### Subtheme 1: concerns about data interpretation, reporting and responsibility

Care-recipients and caregivers had common concerns about the large volume of data generated, and whether that amount of data could be interpreted in a timely and useful manner. As one care-recipient asked: “*… aren’t physicians already totally overwhelmed?”*

Providers were focused on how the sensor data would be collected and reported. As one provider asked *“… would there be a way to easily pull it or, you know, present it in a meaningful way to the team?”* Providers had related concerns regarding who would be responsible for collecting and managing all of the information provided by sensors: *“Well I don’t know who’s going to be responsible for getting all of that information and then figuring out who that gets reported to and how …*”.

##### Subtheme 2: data security

Care-recipients, caregivers, and providers had concerns regarding data security, and how the data would be transmitted. Older adults and caregivers felt that digital data is vulnerable to hacking and release to the public. They expressed specific concerns about particular kinds of sensitive information. A caregiver stated, “*as long as it doesn’t have my social security number on it, I don’t care.”* This suggests that data security should be an important concern in developing sensor technology, and that extra care be taken to protect particular kinds of information such as social security numbers, lab results, or other information that can be abused.

## Discussion

While a number of different studies have investigated the use of sensors to measure phenotypic characteristics of frailty (gait speed, stride length, postural balance, percentage of time spent walking, standing, sitting, etc.), and other studies have assessed older adults’ understanding of frailty [17], there has been only one study of the views of stakeholders regarding the acceptability and adoption of health-related technology solutions for frailty screening and management [18]. The stakeholders in this previous study, conducted in three European countries included frail and robust older adults, their family caregivers, as well as health and social care professionals. Consistent with their findings, we found that the stakeholders in our study all recognized the potential value in the use of sensor technology in frailty screening and monitoring with respect to objectivity and timeliness of data collection and earlier detection of health changes that can result in better health outcomes and cost savings. At the same time, similar concerns were raised regarding affordability, accessibility, and data security. Our study was unique in that patient-caregiver dyads were included to better delineate converging vs. diverging role-dependent views and the use of a diverse pool of medical providers caring for patients with a full spectrum of frailty status improved the generalizability of the opinions.

On the issue of potential benefits, all three groups also focused on the practical benefits of spotting potential hazards in the behavior and lifestyle of older adults, and identifying changes (i.e., moving from a multi-level home with lots of stairs to a single floor dwelling) that would reduce risks and assist the frail older adult and their family to plan for the older adult’s declining health. Providers focused on the potential for sensors to allow them to monitor specific patient activities of concern (such as insomniac behaviors, decreased mobility, and excessive bathroom use) without the need for inpatient admission. In addition, providers felt that sensors could provide objective data that would assist them in documenting the declining health of older adults for their families and caregivers and allow them to better explain the need for support and planning.

Each of the groups also saw potential issues with implementing sensor technologies – mainly data management concerns, which echoed those of other studies of sensor technologies [19]. Common issues included worries that the large volume of data that sensors could produce may complicate data interpretation and communicating the results in a timely and useful fashion to the older adults (and their families). Related concerns were transmission and reporting of data– i.e., data security. Care-recipients and caregivers differed in that the older care-recipients had few concerns that their sensor data could become publicly available, as they assumed that such data (e.g., location tracking on a smartphone) was already publicly known. With respect to data security, providers and caregivers shared concerns that sensor data be protected from release. Accordingly, it will be important to incorporate robust data security features into any such sensor technology to ensure acceptance by caregivers and providers.

Where participants had divergent views, it was usually providers that had different perspectives from caregivers and older adults (who tended to have more similar views). For example, with respect to data management, providers recognized that the sheer volume of sensor data could make it difficult to analyze and incorporate readily into an appropriate care plan for their patients; and they worried about who would be responsible for handling and reporting health information derived from sensor technology. Further, they worried that there was an increased risk and potential liability associated with missing important sensor information, and that they may not be reimbursed for time spent, outside of the clinic, in reviewing and acting on sensor data. This indicates the need for developing systems to process and analyze the sensor data flow to provide information in a form that providers can readily incorporate into care plans. In addition, insurance plans (private and government run) should reimburse providers for the time and financial burden imposed by integrating sensor-derived information into a care plan for older adults.

Caregivers and older adults (but not providers) expressed more concerns about how the use of sensors would impact their personal contact with their healthcare provider. They worried that the implementation of sensor technologies could reduce or supplant their time one-on-one with providers. This suggests that sensor technology would be more acceptable if used as an adjunct to existing healthcare modalities, to enrich and improve the interactions between older adult and healthcare provider.

Regarding affordability and accessibility of new technologies, most sensor technologies rely on internet access in order to convey information from the sensor to its users. About one-third of older adults aged 65 and older never use internet and only 42% report owning smartphones [20], so any implementation of sensor technology may need to include financial support or incentives for older adults without internet access. In addition, caregivers and older adults expressed a preference for unobtrusive sensors similar to Fitbits and smartwatches, indicating that there exists a significant cohort of present day caregivers and care recipients who are less comfortable or adept at using technology, and would benefit from highly user-friendly devices that require little user input. This is consistent with the Pew Research Center survey in 2017 showing that 48% of older adults need help with use of a new electronic device [20]. Therefore, the availability of training opportunities and social support would be the key to increasing adoption of new technologies by older adults and boosting their confidence of becoming more digitally connected. As new generations of older adults are becoming more experienced with diverse technologies and more “tech-savvy,” aging, technology, and healthcare will be inextricably linked in the daily lives of older adults.

The challenge of dealing with the increasing gap between the needs of a fast growing aging population and the available informal and formal care resources has incited great interest in using technology to deliver cost-effective care for the most vulnerable. From the standpoint of care management and delivery, it is critical to minimize health risks (e.g., falls) associated with frailty by making lifestyle adjustments and reducing environmental hazards. It is equally important to implement real life monitoring of health changes in order to prevent or delay the more damaging consequences. It is the latter where new technologies including wearable sensors may have great potential for detecting subclinical changes such as slowing gait speed that may not be captured by routine clinical visits. On the issue of cost-saving, given that older adults make up nearly one quarter [21, 22] of all emergency department visits and approximately 50% of those visits were nonemergent and therefore potentially preventable [23], a good case can be made for the unprecedented opportunity afforded by the advent of sensor technologies for real-time and continuous health monitoring. Enriched by a greater level of granularity, frequency, and sensitivity, the resulting digital data thus hold great promise for early diagnosis of impending health issues that can be handled more effectively by primary care with significant cost-savings. In this sense, the use of technology is meant to be value-added and to complement rather than replace the clinical encounters. However, in order to realize the full potential of technology, major bottlenecks for its wide use in geriatric care will have to be addressed. Besides factors related to stakeholder-specific characteristics such as affordability, accessibility, and perceived usefulness, one major bottleneck for dissemination of new technology in the big data era is data analytics. As pointed out by our study participants, getting data from sensors is cheap; making sense of them with the purpose of informing clinical decision-making is challenging. It is worth noting that the development of data analytics should be informed by theory and its relationship to the target of measurement by the technology. This is particularly important in the field of gerontology and geriatrics where many of the outcomes (also termed “constructs”) such as frailty are not directly observable but can be inferred from multiple measurable indicators of the underlying constructs (e.g., gait speed, grip strength). For this reason, the selection and development of technology and analytics should ensure a good fit between what the technology is capable of measuring and the appropriateness of inference made with respect to the intended construct on the basis of such measurement. In other words, measurement without theory limits the value to science and to policymakers of the results obtained or obtainable by new technologies [24].

### Strengths and limitations

One key strength of our study is the inclusion of multiple key stakeholders with both overlapping and role-specific views on the use of sensor technologies for frailty diagnosis and monitoring. The other strength is recruitment of older adults representing the full frailty spectrum from non-frail to prefrail to frail so that potentially varied opinions by frailty status can be more comprehensively studied. The care-recipient/caregiver dyads also provided the opportunity to explore shared as well as diverging opinions that could be shaped by specific relationship dynamics within dyads. One limitation of our study is that we did not intentionally probe our participants about the psychological impact of being diagnosed with frailty, perceived and self-stigma associated with being frail and its influence on adoption of new technologies. However, a recent study by Schoenborn et al. [16] showed that compared to participants who were non-frail or pre-frail, those who were frail were more receptive to discussing their frailty status with clinicians despite having negative perceptions about the term “frail.” This suggests that the benefits associated with frailty assessment could overcome the stigma of frailty if greater emotional and social support are made available to older adults. Another limitation is that the care-recipients and caregivers in our study were more highly educated which may affect the generalizability of findings.

The present study may not be broadly generalizable, but qualitative studies rarely are because generalizability is not typically their purpose. Since little is presently known about how receptive older adults, their informal caregivers and medical providers are to the use of wearable and/or installed sensors at home to assess frailty, it is helpful to start with qualitative methods to expand the knowledge of this topic. This provides important preliminary information to facilitate the development of more structured studies. In this case, a qualitative understanding of the preferences of older adults with regard to specific types of sensor technologies will allow us to better target sensor technologies that will be more readily adopted by older adults.

## Conclusions

Overall, the perspectives of older care recipients, their caregivers, and medical providers in this study indicated interest and general acceptance of using sensor-based technologies to assess frailty in older adults with care needs. The main concerns were related to voluminous data and strategies for responsible and secure transfer and reporting of data, including its distillation into useful and timely care information. Sensor-based technologies for frailty assessment show promise based on the qualitative findings in this study, but should undergo pilot testing for feasibility and utility. This will inform the larger goal of helping older adults to maintain independence while tracking potential health declines, especially among the most vulnerable, for early detection and intervention.

## Supplementary Information

**Additional file 1.** Interview Guides.

## Data Availability

All data generated and analyzed during the current study, are not publicly available due to the need to protect participant confidentiality.

## Abbreviations

ADL: Activities of daily living
HEP: Hopkins Elder Plus
IADL: Instrumental activities of daily living
PACE: Program for all-inclusive care for the elderly
