# Supplementary material for: Developing a sensor-based mobile application for in-home frailty assessment: a qualitative study
Source: BMC Geriatr. 2021 Feb 4;21:101. doi: 10.1186/s12877-021-02041-z (PMC7863502; doi:10.1186/s12877-021-02041-z)
Supplement: Supplementary file 1 — Additional file 1. Interview Guides. [file 12877_2021_2041_MOESM1_ESM.doc]

**Appendix**

**Interview Guides**

**Older Adults**

Introductions

1. Please tell us your name and your age? (reassure that names are to aid transcription and will not be retained in the transcribed text).
2. Medical professionals define frailty as a chronic health condition, like high blood pressure or diabetes, that can slowly develop over time. People who are frail have a harder time bouncing back or recovering after they get sick or hurt, like from an infection or a surgery. Frailty is more common as people get older but is not part of normal aging; it is not something you always get as you get older. Common signs and symptoms of frailty include weight loss, walking slower, weaker strength, feeling more exhausted, less physical activities.

What questions do you have about what I just said?

Can you think of someone that you know who may be frail?

1. We know that if a Mr. Jones is “frail”, he may be more likely to get weaker over time, or may not live as long, and that he is at higher risk for side effects from certain medications or procedures. But there are things we can do to help Mr. Jones, such as exercise, healthy diet, making sure he’s on the right medications, and avoiding procedures that may be too risky.

Do you think that patients would want to know these risks that come with being frail, like getting weaker more quickly over time, may not live as long, higher risk for side effects? Or do you think doctors should make the appropriate recommendations without discussing these risks?

How would knowing the risks be helpful? Would knowing about the risks make someone more motivated to make a positive change about their health?

1. Have you heard about sensing devices or sensors?

The term sensing device refers to any system that is attached to either the body or an object that measures health relevant information such as physical activity (e.g., steps walked), sleep quality, and heart rate.

The data collected by these sensors can be transmitted to your doctor in real time for the purpose of

a) letting him/her monitor and detect health changes that may flag frailty issues, and

b) developing an individualized care plan intended to minimize the risk of developing adverse outcomes associated with frailty.

1. What do you think about the idea of either having you wear these sensors (e.g., a wristband or watch) or installing in your home? Here are a few example of these sensors [show the pictures].
2. Next, I would like to ask for your opinion toward using of sensors in following areas [Use following questions as probes]
3. How would you feel about wearing a device vs. having sensors installed in your home?
4. Do you have any preference regarding where you would like a sensing device to be worn? (e.g., wrist, arm, abdomen, waist, leg, hip, neck, ankle, etc.)
5. What fears or concerns do you have about using sensors in the home?
6. How long and how often would you be willing to wear a sensor in your home? If it meant you did not have to go to the doctor for a visit, would that influence your willingness?
7. How would you feel about allowing data to be sent away to your doctor and analyzed or stored on a database?
8. Would you use a device that you have to apply yourself without assistance?
9. How interactive would you like to be with the sensors? Do you prefer having the sensors do their own thing with minimal human interaction?
10. How would you feel about wearing a device that would tell the clinician if you did not wear it often enough?
11. Next, I present you a list of features about a sensing device (e.g., ease of use, being discreet). Would you please share with the group the ones that you value the most?

A sensing device that you use should …

… be comfortable

… be portable (light and small)

… be discreet

… be simple to operate and maintain

… give instant feedback

… have clear and readable instructions

… not attached to a person unless needed

… increase the accuracy of current clinical procedures

… motivate the people using it

… not affect normal daily behavior

… reduce travel to clinics and hospitals

1. How would you like to communicate with your doctor regarding the information collected by the sensors?

Would iPad, tablet, or smart phone be a useful tool for you to communicate with your doctor?

And if so, what are your expectations for such communication, anticipated outcomes, and timeframe for receiving feedback from your doctor?

How often would you look at information provided to you directly in an iPad, tablet, or smart phone?

1. Would you willing to use the iPad, tablet, or smart phone to periodically complete short questionnaires about changes in self-care behaviors such as diet, sleep quality, physical activity, and social engagement and share such information with your doctor?
2. Do you need any assistance with using the sensing devices and/or communication devices such as iPad? If yes, what kind of assistance do you need? Do you have someone who can help you?
3. Would you like us to share the information collected by the sensors with your primary caregiver and/or family members?

**Caregiver**

Introductions

1. Please tell us your name and your age? (reassure that names are to aid transcription and will not be retained in the transcribed text).
2. Medical professionals define frailty as a chronic health condition, like high blood pressure or diabetes that can slowly develop over time. People who are frail have a harder time bouncing back or recovering after they get sick or hurt, like from an infection or a surgery. Frailty is more common as people get older but is not part of normal aging; it is not something you always get as you get older. Common signs and symptoms of frailty include weight loss, walking slower, weaker strength, feeling more exhausted, less physical activities.

What questions do you have about what I just said? Can you think of someone that you know who may be frail?

1. We know that if a Mr. Jones is “frail”, he may be more likely to get weaker over time, or may not live as long, and that he is at higher risk for side effects from certain medications or procedures. But there are things we can do to help Mr. Jones, such as exercise, healthy diet, making sure he’s on the right medications, and avoiding procedures that may be too risky.

Do you think that patients would want to know these risks that come with being frail, like getting weaker more quickly over time, may not live as long, higher risk for side effects? Or do you think doctors should make the appropriate recommendations without discussing these risks?

How would knowing the risks be helpful? Would knowing about the risks make the person whom you’re caring for more motivated to make a positive change about his/her health?

1. Have you heard about sensing devices?

The term sensing device refers to any system that is attached to either the body or an object that measures health relevant information such as physical activity (e.g., steps walked), sleep quality, and heart rate. The data collected by these sensors will be automatically transmitted to his/her doctor for the purpose of (a) letting the doctor monitor and detect health changes that may flag frailty issues, and (b) developing an individualized care plan for your _______ [care recipient] intended to minimize the risk of developing adverse outcomes associated with frailty.

1. What do you think about the idea of either having your _____ [care recipient] wear these sensors (e.g., a wristband or watch) or installing in his/her home? (show pictures of different types of sensors)
2. Next, I would like to ask for your opinion toward using of sensors in following areas [Use following questions as probes]
3. How would you feel about letting your _________wear a device vs. have sensors installed in his/her home?
4. Do you have any preference regarding where you would like a sensing device to be worn by your _______? (e.g., wrist, arm, abdomen, waist, leg, hip, neck, ankle, etc.)
5. What fears or concerns do you have about using sensors in the home?
6. How long and how often would you be willing to let your _________ wear a sensor in his/her home? If it meant your _________ did not have to go to the doctor for a visit, would that influence your willingness?
7. How would you feel about allowing data to be sent away to your ______’s doctor and analyzed or stored on a database?
8. How interactive would you like to be with the sensors? Do you prefer having the sensors do their own thing with minimal human interaction?
9. Would you be willing to provide assistance to your ________ when using such devices? What type of assistance are you willing to perform?
10. Next, I present you a list of features about a sensing device (e.g., ease of use, being discreet). Would you please share with the group the ones that you value the most as a caregiver?

A sensing device that you use should …

… be comfortable

… be portable (light and small)

… be discreet

… be simple to operate and maintain

… give instant feedback

… have clear and readable instructions

… not attached to a person unless needed

… increase the accuracy of current clinical procedures

… motivate the people using it

… not affect normal daily behavior

… reduce travel to clinics and hospitals

1. How would you like to communicate with your doctor regarding the information collected by the sensors?

Would iPad, tablet, or smart phone be a useful tool for you to communicate with your ______’s doctor?

and if so, what are your expectations for such communication, anticipated outcomes, and timeframe for receiving feedback from his/her doctor?

1. Do you anticipate any assistance that your ______ may need with using the sensing devices and the iPad, tablet, or smart phone?

If yes, what kind of assistance can you provide?

1. Would you like to have your _______ share with you the information collected by the sensors or the communication between your _____ and his/her doctor?

**Medical Team**

Introductions

1. Please tell us your name and your primary role in the care team? (reassure that names are to aid transcription and will not be retained in the transcribed text).
2. Medical professionals define frailty as a chronic health condition, like high blood pressure or diabetes, that can slowly develop over time. People who are frail have a harder time bouncing back or recovering after they get sick or hurt, like from an infection or a surgery. Frailty is more common as people get older but is not part of normal aging; it is not something you always get as you get older. Common signs and symptoms of frailty include weight loss, walking slower, weaker strength, feeling more exhausted, less physical activities. Frailty, multimorbidity and disability are overlapping but yet distinct entities. For example, you can have someone who is disabled but not frail and vice versa.

What questions do you have about what I just said?

1. We know that if a Mr. Jones is “frail”, he may be more likely to get weaker over time, or may not live as long, and that he is at higher risk for falls, or side effects from certain medications or procedures.

How would knowing the risks be helpful to you as a medical professional?

Would knowing about the risks make you change the care plan of your patient in any way?

If yes, what changes would you consider making?

1. Have you heard about sensing devices?

The term sensing device refers to any system that is attached to either the body or an object that measures health relevant information such as physical activity (e.g., steps walked), sleep quality, and heart rate.

1. What do you think about the idea of either having your patients wear these sensors (e.g., a wristband or watch)?

or installing them in his/her home?

1. What could be the benefits of using such in-home monitoring system?
2. What could be the negative consequences of using such in-home monitoring system?
3. How would the use of such system affect your workflow and/or workload specifically? What resources would you need?
4. Are there any specific health related information about your patient that you wish you could monitor at home?

How would you plan to use such information?

1. How would you like to communicate with your patient regarding the information collected by the sensors?

Would it be acceptable to have the data collected by these sensors automatically transmitted to you 24/7 via a mobile app?

If yes, what are your expectations for such communication, anticipated outcomes, and timeframe for providing feedback to your patient?
